# CT radiomics-based model for predicting TMB and immunotherapy response in non-small cell lung cancer

**DOI:** 10.1186/s12880-024-01221-8

**Published:** 2024-02-15

**Authors:** Jiexiao Wang, Jialiang Wang, Xiang Huang, Yanfei Zhou, Jian Qi, Xiaojun Sun, Jinfu Nie, Zongtao Hu, Shujie Wang, Bo Hong, Hongzhi Wang

**Affiliations:** 1 School of Basic Medical Sciences, Anhui Medical University, Hefei, Anhui China; 2 Anhui Province Key Laboratory of Medical Physics and Technology, Institute of Health and Medical Technology, Hefei Institutes of Physical Science, Chinese Academy of Sciences, Hefei, Anhui China; 3 Hefei Cancer Hospital, Chinese Academy of Sciences, Hefei, Anhui China; 4 University of Science and Technology of China, Hefei, Anhui China

**Keywords:** Non-small cell lung cancer, Tumor mutation burden, Immunotherapy, Radiomics, Computed tomography

## Abstract

**Background:**

Tumor mutational burden (TMB) is one of the most significant predictive biomarkers of immunotherapy efficacy in non-small cell lung cancer (NSCLC). Radiomics allows high-throughput extraction and analysis of advanced and quantitative medical imaging features. This study develops and validates a radiomic model for predicting TMB level and the response to immunotherapy based on CT features in NSCLC.

**Method:**

Pre-operative chest CT images of 127 patients with NSCLC were retrospectively studied. The 3D-Slicer software was used to outline the region of interest and extract features from the CT images. Radiomics prediction model was constructed by LASSO and multiple logistic regression in a training dataset. The model was validated by receiver operating characteristic (ROC) curves and calibration curves using external datasets. Decision curve analysis was used to assess the value of the model for clinical application.

**Results:**

A total of 1037 radiomic features were extracted from the CT images of NSCLC patients from TCGA. LASSO regression selected three radiomics features (Flatness, Autocorrelation and Minimum), which were associated with TMB level in NSCLC. A TMB prediction model consisting of 3 radiomic features was constructed by multiple logistic regression. The area under the curve (AUC) value in the TCGA training dataset was 0.816 (95% CI: 0.7109–0.9203) for predicting TMB level in NSCLC. The AUC value in external validation dataset I was 0.775 (95% CI: 0.5528–0.9972) for predicting TMB level in NSCLC, and the AUC value in external validation dataset II was 0.762 (95% CI: 0.5669–0.9569) for predicting the efficacy of immunotherapy in NSCLC.

**Conclusion:**

The model based on CT radiomic features helps to achieve cost effective improvement in TMB classification and precise immunotherapy treatment of NSCLC patients.

**Supplementary Information:**

The online version contains supplementary material available at 10.1186/s12880-024-01221-8.

## Introduction

Lung cancer is the leading cause of cancer-related deaths worldwide [1]. Non-small cell lung cancer (NSCLC) accounts for about 85% of all lung cancers. Lung adenocarcinoma (LUAD) and lung squamous cell carcinoma (LUSC) are the most predominant types of NSCLC [2]. Patients with early-stage NSCLC can be treated by surgical resection, but about 75% of patients are already in advanced stages when they are first diagnosed [3]. Although progress on molecular targeted therapy and immunotherapy has been made to substantially improve the survival of advanced NSCLC, the overall 5-year survival rate is still low (~ 20%) [4]. For NSCLC patients harboring actionable mutations (EGFR, ALK or ROS1 etc.), targeted therapy is more effective than other therapies [5]. However, for the selection of NSCLC patients responsive to immunotherapy, there is no effective, easily-detectable and low-cost predictive biomarker.

Despite rapid advances of immunotherapy in NSCLC, only a minority of patients respond to immune checkpoint blockade with anti-PD-1 and PD-L1 antibodies. PD-L1 expression detected by immunohistochemistry (IHC), and tumor mutational burden (TMB) measured by next generation sequencing (NGS) are the best-studied biomarkers for the response to immunotherapy in NSCLC [6]. For NSCLC patients whose tumors have high PD-L1 expression, overall survival (OS) and progression-free survival (PFS) of patients treated by immune checkpoint inhibitor are thought to be superior to first-line chemotherapy regimens [7]. But a previous study has indicated that only 44.8% of NSCLC patients achieved an objective response when treated with PD-L1 antibody pembrolizumab monotherapy, even in a highly selected patient population (PD-L1 expression ≥50%) [8]. Another study showed that for NSCLC patients (PD-L1 expression ≥5%), median PFS was 4.2 months for patients treated with PD-1 antibody nivolumab and 5.9 months for patients treated with chemotherapy [9]. These studies show that high PD-L1 expression could not be an effective biomarker to delineate the beneficiary population for immunotherapy.

TMB is defined as the total number of somatic non-synonymous mutations detected per million bases [10]. If tumors have more somatic non-synonymous mutations that are transcribed and translated with more neoantigens that the body does not recognize, these neoantigens will activate T lymphocytes and other relevant immune cells. Therefore, TMB is considered a biomarker of immune response to PD-1/PD-L1 inhibitors in NSCLC patients [11]. Studies have indicated that NSCLC patients with higher TMB had a better prognosis compared to those with relatively lower TMB, when treated with immunotherapy. Pan et al. demonstrated a median OS of 18 months for NSCLC patients (TMB ≥ 10) treated with pembrolizumab (11 months for TMB < 10) [12]. Carbone et al. also indicated that NSCLC patients with high TMB treated with nivolumab had a median PFS of 9.7 months, which was longer than those treated with chemotherapy (5.8 months) [9]. Another study showed that for NSCLC patients with high TMB (TMB ≥ 10) and negative PD-L1 expression, patients treated with nivolumab in combination with ipilimumab had longer median PFS than those treated with chemotherapy (7.7 months vs 5.3 months) [13]. The PD-1 monoclonal antibody pembrolizumab has been approved by the US Food and Drug Administration (FDA) for the treatment of solid tumors with high TMB levels [14] . Although TMB is thought to predict the response to PD-1/PD-L1 blockade in NSCLC patients, TMB needs to be accurately calculated by whole exome sequencing (WES), a method that is expensive and unaffordable for most patients [15]. It may be impractical for clinical use because clinical samples are not available for advanced inoperable patients [16]. With the development of second-generation sequencing, although panel-based sequencing of tumor tissues is common in clinical practice, there are differences in panel size [17]. Therefore, it is of clinical significance to develop non-invasive and cost-effective predictive biomarkers for immunotherapy in NSCLC.

The radiomics technique is inexpensive, non-invasive, time-consuming and easy to perform, overcoming the above-mentioned shortcomings [18]. The images obtained from computed tomography (CT) scans show a potential correlation between deep tumor features and TMB status that can be quantitatively analyzed [19]. In addition, the machine learning approaches can link the molecular and imaging characteristics of patients’ tumors [20]. Therefore, in this study, a model to predict TMB levels in NSCLC patients was developed by using CT images-based radiomics techniques, and the predictive value of this model for the response of immunotherapy in NSCLC patients was also evaluated.

## Methods

### Study population and image acquisition

**Fig. 1:**
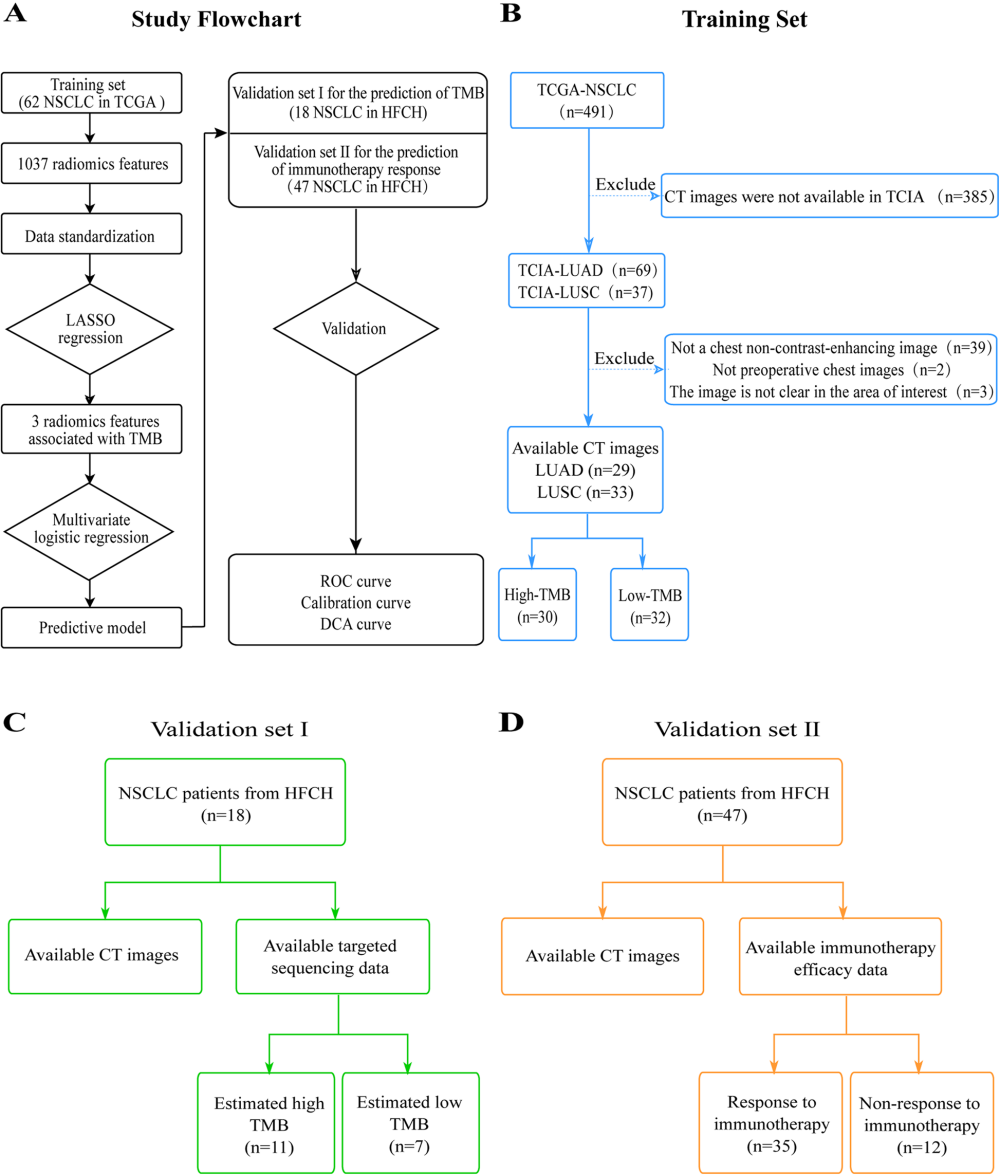
The study workflow and datasets. **A** The study workflow. **B** The training sets. The preoperative chest CT images and TMB data are available in 62 NSCLC patients from TCGA. **C** Validation set I. The preoperative chest CT images and estimated TMB data are available in 18 NSCLC patients from Hefei Cancer Hospital (HFCH), Chinese Academy of Sciences. **D** Validation set II. The preoperative chest CT images and immunotherapy response data are available in 47 NSCLC patients from Hefei Cancer Hospital (HFCH), Chinese Academy of Sciences

This retrospective study included three datasets, a training dataset and two external validation datasets. The training dataset was NSCLC patients from The Cancer Genome Atlas (TCGA). Chest CT images of NSCLC patients from TCGA were downloaded from The Cancer Imaging Archive database (TCIA) [21]. TMB and clinical data of NSCLC patients with same TCIA patient identifiers were downloaded from TCGA database [22]. The inclusion criteria were: 1) pathological diagnosis of NSCLC; 2) preoperative CT scan of the chest and good quality of preoperative CT images of the chest; 3) CT images of the chest in the non-contrast-enhanced period; 4) TMB information can be obtained. Finally, 62 eligible NSCLC patients from the TCIA-LUAD and TCIA-LUSC cohorts were selected as the training set (Fig. 1A and B). Using the same CT image inclusion criteria, 18 and 47 NSCLC patients were recruited from the Hefei Cancer Hospital (HFCH), Chinese Academy of Sciences as validation set I and validation set II, respectively. NSCLC patients in validation set I had the targeted NGS sequencing, and NSCLC patients in validation set II had the immunotherapy from July 31, 2021-August 30, 2022. All CT images were retrieved from the Picture Archiving and Communication System (PACS; CAREstream Medical Ltd.) and all data were stored in Digital Imaging and Communications in Medicine (DICOM) format.

### CT imaging parameters

In the training set, non-contrast enhanced CT images of the chest were obtained from three different manufacturers: Philips (https://www.philips.com.cn/healthcare), General Electric (GE, https://www.gehealthcare.cn) and Siemens (https://www.siemens-healthineers.cn) Medical Systems, respectively. In the validation set, CT images were acquired using a 256-layer Brilliance iCT scanner from Philips. CT image scanning parameters: tube voltage, 110–120 kV; tube current, 100–150 mAS; rotational speed, 0.5 s; reconstructed slice thickness, 1–5 mm; matrix, 512*512; kernel function, standard.

### Region of interest segmentation and features extraction

The CT images were imported into the 3D-Slicer software (version 5.1.0, https://www.slicer.org/) [23] and read using a longitudinal window (window width 400, window position 40). The image dataset was very diverse in terms of manufacturers, scanning parameters (modes), etc. In order to standardize the images, we reconstructed the CT images using a soft tissue algorithm. The resampling voxel size was 3*3*3 mm and LoG kernel size was 4*5 mm. A respiratory physician and a thoracic surgeon used the software’s built-in mapping tool to manually delineate the location of tumors in the training and validation sets, respectively. The regions of interest (ROI) of all images were checked by a radiologist. If there was an obvious inconsistency of opinions between the three observers, an agreement was reached through a discussion. Then, the 3D reconstruction function of the software was used to reconstruct the segmented 2D ROI into a 3D stereo state. This study used coronal, sagittal and cross-sectional reconstructed images. Next, radiomics features were extracted from the ROI using the built-in plug-in of 3D-Slicer software, SlicerRadiomics (version a57d142) [23]. The radiomics features were classified into four catalogues as follows: 1) morphological features; 2) first-order grayscale histogram features; 3) second-order and higher-order texture features; and 4) wavelet-based features. All radiomics features were normalized by z-score, and mapped to around 0.

### Calculation of TMB

In the training set, the NSCLC tumors of TCGA samples were sequenced by WES. TMB was calculated as the number of somatic nonsynonymous mutations divided by the full exon chip size (38 Mb). Based on the median TMB of all samples, all patients were divided into a high TMB cohort (*n* = 30, TMB > 6.711, range 6.842–25.500) and a low TMB cohort (*n* = 32, TMB < 6.711, range 0.526–6.711).

In the validation set I, the tumor samples of NSCLC patients were subjected to NGS using cancer-related targeted genes panel. TMB was estimated by the number of base mutations per megabase, which was calculated by the number of somatic nonsynonymous mutations divided by total panel size of target sequencing region (0.26 Mb).

### The determination of immunotherapy response

In validation set II, each patient was treated by immunotherapy with PD-L1 inhibitor tislelizumab. Immunotherapy response was assessed by an experienced physician, according to the iRECIST criteria. Immune complete response (iCR), immune partial response (iPR) and immune stable disease (iSD) were defined as response to immunotherapy. Immune confirmed progression (iCPD) was defined as non-response to immunotherapy [24].

### Feature selection and radiomics feature model construction

In order to keep the parameters as simple as possible while ensuring the best fit error and making the model generalizable, the least absolute shrinkage and selection operator (LASSO) regression was used to perform dimensionality reduction on high-dimensional data and select radiomics features as independent TMB predictors. Wilcoxon rank-sum test was used to show the potential association between selected radiomics features and TMB levels. Finally, a multivariate logistic regression algorithm was used to construct a model to predict TMB level. The model was presented in the form of a nomogram. Variance inflation factors (VIF) were calculated to determine whether each independent predictor has multicollinearity.

### Model evaluation and statistical analysis

The receiver operating characteristic (ROC) curve was plotted and the area under the curve (AUC) was calculated to evaluate the accuracy of the model prediction. The maximum point of the Youden index (ie, sensitivity + specificity - 1) was used to define the optimal threshold of the ROC curve. Sankey energy shunt diagrams were drawn online using the BioLadder bioinformatics cloud platform (https://www.bioladder.cn/web/#/chart/59). Calibration curve was used to determine the agreement between predictions and observations. A decision curve analysis (DCA) was performed to observe the overall net benefit of the prediction model to assess clinical usability. The Pearson’s Chi-squared test, Fisher’s exact test and Wilcoxon rank sum test were used to determine whether there was a significant difference between data that obeyed or did not obey a normal distribution (two-sided *p*-value< 0.05). The statistical analyses and machine learning algorithms involved in this study were performed using R software (Version: R 4.1.3; http://www.R-project.org).

## Result

### The clinical characteristics of NSCLC patients

The flowchart of this study was shown in Fig. 1A. The clinicopathological features of all NSCLC patients from the training dataset and the two validation datasets were shown in Table 1. In the TCGA training dataset, non-contrast enhanced CT images of the chest and TMB values were available in 62 NSCLC patients. The WES data of 62 NSCLC tumors were downloaded from the TCGA database and their TMB values were calculated, and the median TMB value of all samples was 6.711. The median TMB was used as a criterion for dichotomous classification. When the TMB was greater than 6.711, it was considered high TMB, otherwise it was considered low TMB (Fig. 1B).

**Table 1:** Clinicopathological features of NSCLC patients in the training and validation datasets

Characteristics	Overall *N* = 127	Training set *N* = 62	Validation I set *N* = 18	Validation II set *N* = 47
**Gender**				
Female	34 (27%)	24 (39%)	6 (33%)	4 (8.5%)
Male	79 (62%)	24 (39%)	12 (67%)	43 (91%)
Unknow	14 (11%)	14 (22%)	0 (0.0%)	0 (0.0%)
**Clinical T stage**				
T1-T2	54 (43%)	39 (63%)	7 (39%)	8 (17%)
T3-T4	39 (31%)	9 (14%)	8 (44%)	22 (47%)
Unknow	34 (27%)	14 (23)	3 (17%)	17 (36%)
**Clinical N stage**				
N0-N1	50 (39%)	40 (64%)	5 (28%)	5 (11%)
N2-N3	44 (35%)	8 (13%)	11 (61%)	25 (53%)
Unknow	33 (26%)	14 (23%)	2 (11%)	17 (36%)
**Clinical M stage**				
M0	57 (45%)	39 (63%)	2 (11%)	16 (34%)
M1	29 (23%)	2 (3.0%)	13 (72%)	14 (30%)
Unknow	41 (32%)	21 (34%)	3 (17%)	17 (36%)
**Clinical TNM stage**				
Stage I-II	33 (26%)	31 (50%)	0 (0.0%)	2 (4.0%)
Stage III-IV	68 (54%)	16 (26%)	15 (83%)	37 (79%)
Unknow	26 (20%)	15 (24%)	3 (17%)	8 (17%)
**Age, median (range)**	66 (39–84)	66 (39–84)	64 (50–79)	66 (54–79)
**Histology**				
LUAD	56 (44%)	19 (31%)	18 (100%)	19 (40%)
LUSC	57 (45%)	29 (47%)	0 (0.0%)	28 (60%)
Unknow	14 (11%)	14 (22%)	0 (0.0%)	0 (0.0%)

In Validation Set I, non-contrast enhanced CT images of the chest were available in 18 NSCLC patients from Hefei Cancer Hospital (HFCH), Chinese Academy of Sciences. The tumor samples of NSCLC patients were subjected to targeted NGS detection. The estimated TMB was calculated by the number of somatic nonsynonymous mutations divided by total panel size of target sequencing region (0.26 Mb). The estimated median TMB value of all samples was 11.5. The median TMB was used as a criterion for dichotomous classification. When the estimated TMB was greater than 11.5, it was considered high TMB, otherwise, and when the estimated TMB was less than 11.5, it was considered low TMB (Fig. 1C).

In validation set II, non-contrast enhanced CT images of the chest were available in 47 NSCLC patients from Hefei Cancer Hospital (HFCH), Chinese Academy of Sciences. The 47 NSCLC patients were subjected to immune checkpoint inhibitor therapy. 35 patients responded to immunotherapy, and the remaining patients did not respond to immunotherapy (Fig. 1D).

### Selection of radiomics features associated with TMB levels

**Fig. 2:**
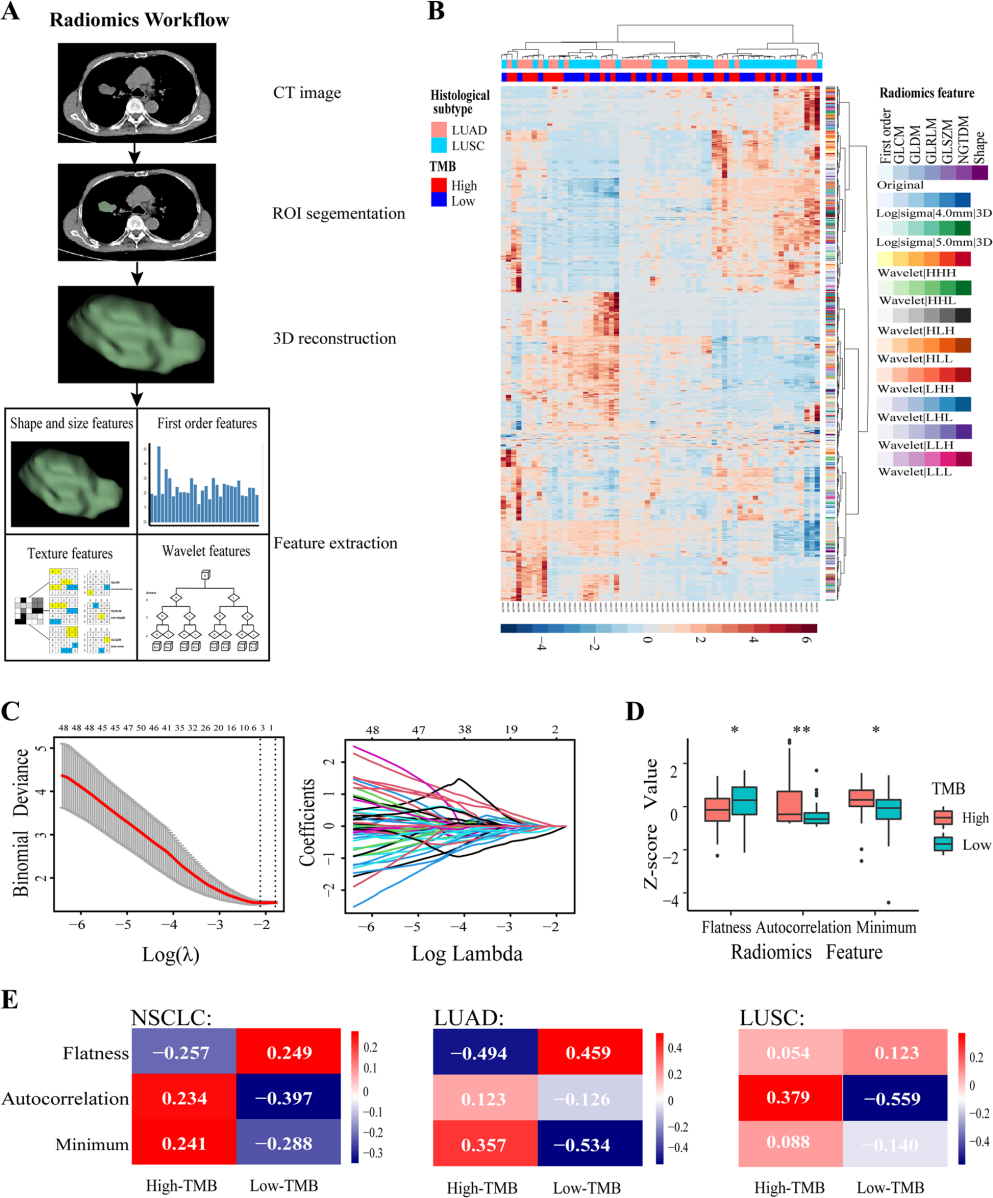
Three TMB-associated radiomics features are selected. **A** Radiomics workflow for the study. The ROI of the tumor is segmented and reconstructed to extract high-dimensional radiomics features. **B** The unsupervised clustering heatmap shows all the radiomic features extracted from the tumor ROI of 62 NSCLC patients in the training set. **C** The LASSO regression was used to select the radiomics features associated with TMB. The left panel shows the tuning parameter (λ) of the LASSO regression model selected by the 10-fold cross-validation method based on the minimum criterion. The right panel shows the LASSO coefficient profile consisting of 1037 radiomics features. The dashed vertical upper x-axis represents the average number of radiomics features and the dashed vertical lower x-axis corresponds to a log(λ) value of − 2.117. **D** The Z-score values for three features of NSCLC patients between high TMB and low TMB. **E** The heatmap of the correlation between radiomics features and TMB in the training set

Figure 2 a illustrated the radiomics workflow for this study. In the TCGA training dataset (*n* = 62), we segmented the tumor areas at each layer of the NSCLC patient’s CT images. The tumor areas were manually outlined using 3D Slicer software, and then reconstructed in three dimensions (Fig. 2A). A total of 1037 different radiomics features were extracted from the tumor simulation images (Supplementary Table 1). These 1037 radiomics features were divided into six major categories, including first-order, gray level co-occurence matrix (GLCM), gray level dependence matrix (GLDM), gray level run-length matrix (GLRLM), gray level size zone matrix (GLSZM) and neighbouring gray tone difference matrix (NGTDM) (Fig. 2B).

We then used LASSO regression algorithm to select the radiomics features, which were associated with TMB level (Fig. 2C). The three radiomics features, including Flatness (shape of original feature), Autocorrelation (GLCM) and Minimum (first order of wavelet features), were the most associated with TMB levels. The three radiomics features exhibited significant differences between high and low TMB groups (Fig. 2D).

In Fig. 2E, the three radiomics features showed significant correlations between high and low levels of TMB in all NSCLC samples. In LUAD, Flatness (shape of original feature) exhibited a strong negative correlation with the TMB level, and Minimum (first order of wavelet features) exhibited a strong significant positive correlation with the TMB level. On the contrary, Autocorrelation (GLCM) showed a strong positive correlation in LUSC (Fig. 2E). These results suggest that, for the TMB correlation, Flatness (shape of original feature) and Minimum (first order of wavelet features) have a greater contribution in LUAD, while Autocorrelation (GLCM) has a greater contribution in LUSC. Therefore, the three radiomics features were used to build a TMB predictive model in NSCLC.

### Development and validation of the TMB predictive model

**Fig. 3:**
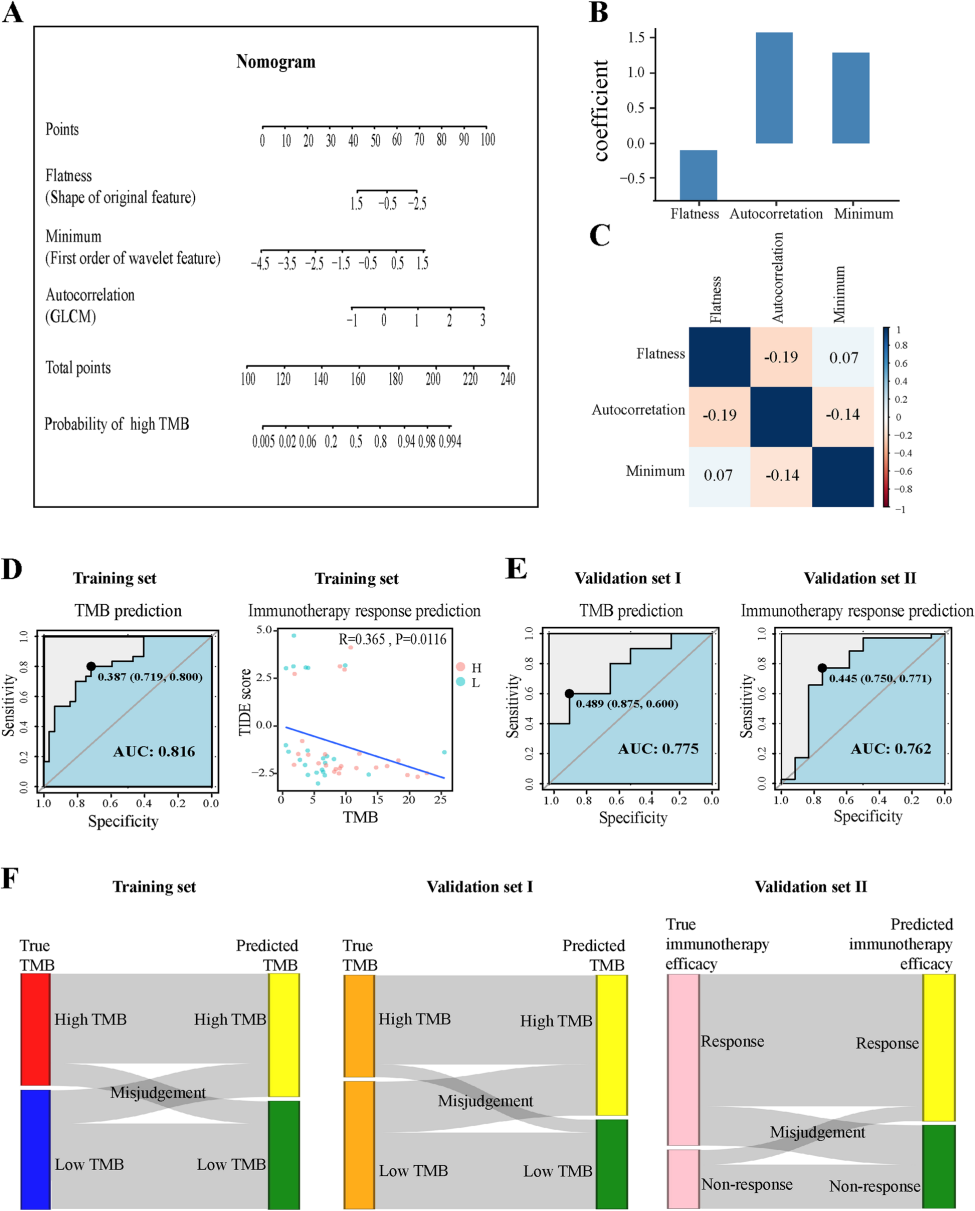
Development of the radiomics model and its performance. **A** The radiomics dynamic nomogram was constructed using the three radiomics features, Flatness (shape of original feature), Minimum (first order of wavelet features) and Autocorrelation (GLCM). **B** Histogram of feature weights for logistic regression. **C** Pearson’s rank correlation among the three radiomic features. **D** ROC curve of the CT-based nomogram to predict TMB in the training set (Left panel). The spearman correlation test between TMB values and TIDE scores. Red dots indicate the NSCLC samples predicted high TMB by the radiomics model. Blue dots indicate the NSCLC samples predicted low TMB by the radiomics model. **E** ROC of the radiomics predictive model in two validation sets. **F** The Sankey diagram shows the patients correctly and incorrectly classified by the radiomics prediction model in the training dataset and two validation datasets

Using the three radiomics features, we built a TMB predictive model by a multivariate logistic regression algorithm. The model was presented as a nomogram (Fig. 3A). The regression coefficients of the three independent variables of the model were shown in Fig. 3B, with larger coefficients indicating its greater weight in the model. These three radiomics features ranked the degree of influence on the prediction model as Autocorrelation, Minimum and Flatness. The VIF of Flatness, Autocorrelation and Minimum were 1.098090, 1.500746 and 1.600343, respectively, indicating that there was no multicollinearity among them. The low Pearson’s correlation analysis indicated that there was no over-fitting and interaction among the three radiomics features (Fig. 3C).

We then evaluated the predictive power and accuracy of the model according to ROC curve analysis. In the TCGA training dataset, the model demonstrated good predictive power with an AUC of 0.816 (95% CI: 0.7109–0.9203). The optimal predictive probability cutoff value for TMB classification (High or Low) was 0.387 (specificity: 71.9%, sensitivity: 80.0%) based on the maximum Youden index (Fig. 3D). In order to show whether the model may predict the response of immunotherapy, TIDE scores were calculated for tumor samples of NSCLC patients in the TCGA training set to assess the potential clinical efficacy of immunotherapy [25]. The spearman correlation test revealed a significant negative correlation between TIDE scores and TMB levels (R = 0.365, *P* < 0.05) (Fig. 3D), indicating that as TMB levels increased, the TIDE scores decreased and the efficacy of immunotherapy increased. The data suggest that NSCLC patients with predicted low TMB are more likely to benefit from immunotherapy.

In the validation set I, the model was used to predict the TMB with an AUC of 0.775 (95% CI: 0.5528–0.9972, cutoff: 0.489), indicating its satisfactory TMB classification capacity (Fig. 3E). Since higher TMB levels have a better response to immune checkpoint inhibitor treatment [26], the model was used to predict the efficacy of immunotherapy. In the validation set II, the radiomics model exhibited a high AUC value of 0.762 (95% CI: 0.5669–0.9569, cutoff: 0.445) to predict the response of immunotherapy, with the sensitivity of 77.1% and specificity of 75.0% (Fig. 3E). The classification results for the training and two validation datasets are presented in the Sankey diagram (Fig. 3F). Therefore, the radiomics model corrects the overfitting problem and demonstrates good discrimination and satisfactory performance for predicting TMB and the response of immunotherapy.

### Clinical usefulness of the radiomics predictive model

**Fig. 4:**
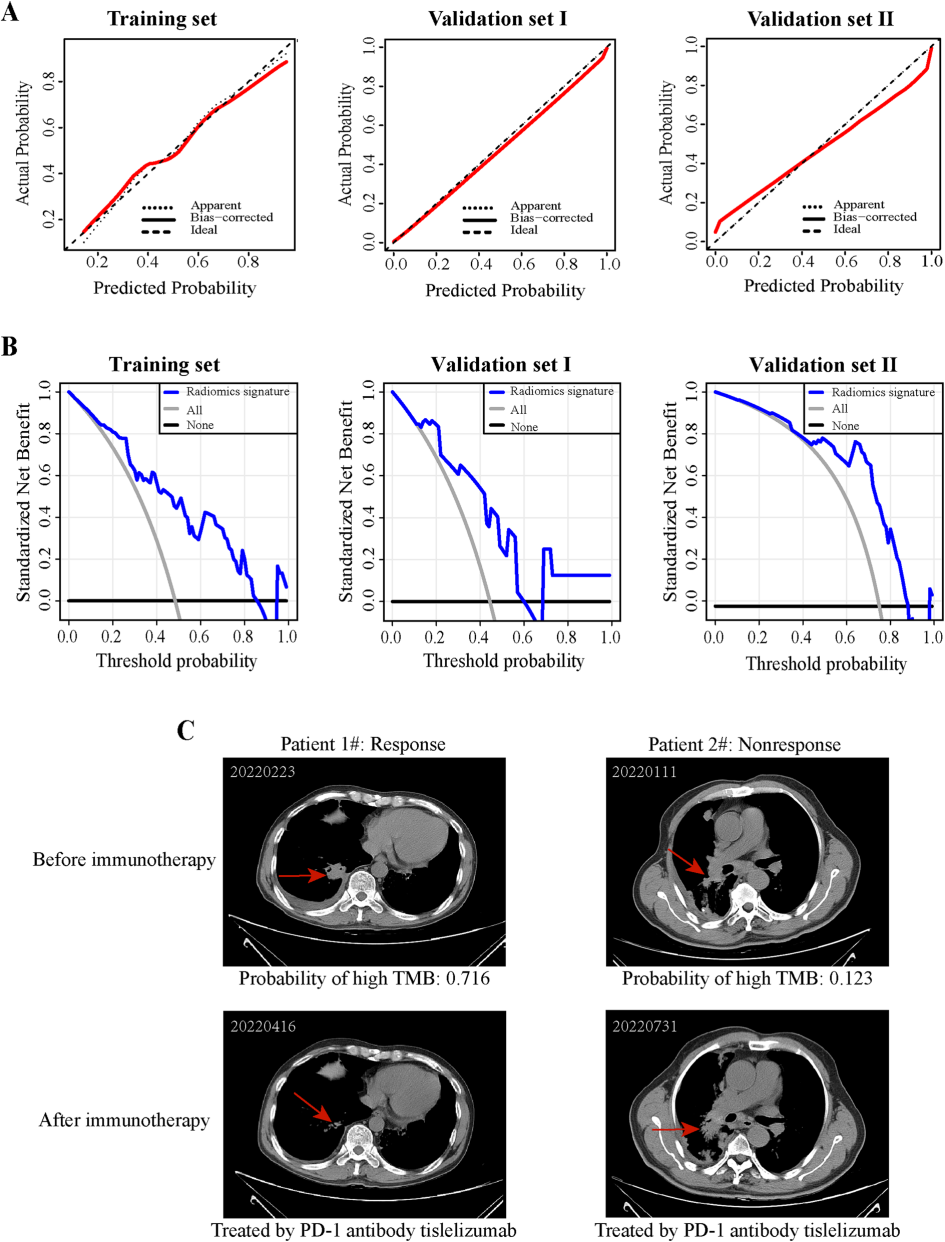
The potential for clinical applications of the radiomics model. **A** Calibration curve of radiomics prediction model in the training set and two validation sets. The solid red line is bias correction by bootstrapping (1000 replicates), indicating the observed radiomics prediction model performance. **B** DCA of radiomics prediction model. The horizontal axis is the threshold probability and the vertical axis is the standard net benefit. **C** CT images of patients who respond and do not respond to immunotherapy. The upper panel shows the CT image before immunotherapy and the lower panel shows the CT image after receiving a certain course of immunotherapy. Probabilities of high TMB predicted by the radiomics model are indicated

The calibration curves of the radiomics predictive model in both the training set and validation sets were shown in Fig. 4A. The predicted probabilities of the classification model were demonstrated to be very close to the actual observed probabilities in the training set and two validation sets (Fig. 4A). The decision curve analysis (DCA) showed that the net benefit of intervening in clinical use for any range of threshold probabilities was better than either treat-all-patients or treat-none-patients strategies in the training set and validation sets (Fig. 4B). As shown in Fig. 4C, patient 1# who responded immunotherapy was predicted to be high TMB and responsive to immunotherapy (predictive probability: 0.761). Patient 2# who did not respond immunotherapy was predicted to be low TMB and non-responsive to immunotherapy (predictive probability: 0.123). The results demonstrate that the model has clinical utility and can help clinicians make better clinical decisions.

## Discussion

As the treatment of tumors enters the era of immunotherapy, immunotherapy is playing an increasingly important role in NSCLC. However, the efficiency of immunotherapy (mainly immune checkpoint inhibitors) in unselected populations is relatively low, and only a small proportion of patients can benefit from immunotherapy [27]. Therefore, our study developed a radiomics models based on preoperative CT images to efficiently predict TMB levels and immunotherapy response in NSCLC patients.

Our study demonstrated that the radiomics model had high AUC values in training set (AUC = 0.816) and two validation sets (AUC = 0.775 and AUC = 0.762), suggesting that it is a reliable model for identifying NSCLC patients who may benefit from immune checkpoint inhibitor therapy. A previous study in advanced NSCLC used five radiomic features to construct a model to predict TMB status with the AUC values of the training set and the validation set, 0.795 and 0.731, respectively [19]. He et al. combined deep learning and CT images to develop a model that can effectively distinguish high and low TMB groups in advanced NSCLC. The AUC values for the training and test cohorts reached 0.85 and 0.81 [20]. He et al. developed a CT-based radiomics model to predict the response of immunotherapy in patients with advanced NSCLC by building a deep learning network. The AUCs of prediction performance were 0.81 and 0.78 in the training and test cohorts, respectively [28]. Compared with these previous studies that used a larger number of features for establishing a model, our model was built using only three radiomics features. This reduces the effect of overfitting issue. Furthermore, a previous study by Yang et al. investigated the association of intra-tumor and peri-tumor areas with TMB level in CT images of NSCLC. The study found that nine radiomic features in intra-tumor area was associated with TMB, while only one radiomic features in peri-tumor area was associated with TMB [29]. Therefore, radiomic features in intra-tumor area were used to construct TMB predictive model in our study. Moreover, a previous study used PET/CT images of NSCLC patients to establish a radiomics model, which was able to predict TMB level [30]. However, a few patients are required to perform PET/CT imaging in clinic, due to its high cost. In addition, previous studies have use the CT images of pulmonary nodules to construct a model to predict TMB level in early stage patients with resectable NSCLC [31, 32]. Generally, the determination of TMB is to predict the prognosis for early stage patients with resectable NSCLC, who are usually not treated by immunotherapy. In a word, our and previous studies indicate that the radiomics model is an efficient, non-invasive and convenient tool to predict TMB and immunotherapy response in NSCLC.

Our study indicated that Flatness (shape of original feature), Autocorrelation (GLCM) and Minimum (first order of wavelet features) were associated with TMB levels in NSCLC. Flatness (shape of original feature) is the feature of tumor morphology, which shows the inconsistent degree of each part of the tumor. For example, the edges of the lesions may be uneven and lobulated. The fine and short burrs, spiny protrusions and jagged changes may be at the edges of the lesions. Autocorrelation (GLCM) shows the similarity between the grey levels of the ROIs. Our study shows that Autocorrelation (GLCM) is positively correlated with TMB, implying that high TMB may be associated with uniform density in tumors. The Minimum (first order of wavelet feature) indicates the minimum value of the grey scale value in the ROIs, implying that TMB may be associated with the presence of the necrosis region of tumors [33]. A previous study showed a significant difference between vacuole sign and TMB status in CT image morphology. This is consistent with our conjecture [19]. Thus, tumor TMB level is reflected in radiomic characteristics. However, the biological mechanisms of the relationships between TMB and radiomic characteristics remain largely unexplained and need to be further explored.

An important issue for the diagnostic models is the potential clinical application. This study performed a decision curve analysis to assess the overall net benefit, which can further show that our prediction model can provide guidance to clinicians. The advantage of the model is not only that the data is relatively easy to obtain, but also that it is non-invasive for patients. Nomogram can visualize each patient’s overall score, providing guidance for clinicians to choose the right treatment decision. Therefore, CT-based biological predictive models can serve as a non-invasive, reliable and easily accessible tool for distinguishing high and low TMB to guide immune checkpoint inhibitors treatment.

Our study also had some limitations. First, this is a retrospective study with a relatively small sample size. This study was only validated in Chinese patients based on a single medical center. Further validation is required in large-sample, multi-center, multi-ethnic prospective randomized clinical trials. Secondly, manual segmentation of the region of interest by doctors is time-consuming and labor-intensive, and an algorithm for automatic segmentation should be developed in the future. Finally, the biological mechanism behind radiomics prediction of TMB levels in NSCLC remains unexplained and requires further study.

## Conclusion

We first found that three radiomic features of pre-treated CT imaging were associated with TMB levels in NSCLC patients. Second, based on the three radiomics features, we developed and validated a model to predict TMB and immunotherapy response. The model could be developed as a non-invasive, reliable, and fast tool to assist clinical decision-making for immunotherapy in NSCLC.

### Supplementary Information

**Additional file 1 Supplementary Table 1.** All the radiomics features extracted from the CT images of 62 NSCLC patients in the TCGA training dataset. The columns of the table (from column C to column AMY) represent different categories of radiomics features. The rows of the table (from row 2 to row 63) represent 62 NSCLC patients.

## Data Availability

The training dataset generated and/or analysed during the current study is available in the TCGA dataset (http://cancergenome.nih.gov) and TCIA dataset (http://www.cancerimagingarchive.net). The validation datasets generated and/or analysed during the current study are not publicly available due to the ethical and patient privacy regulations, but are available from the corresponding author (Bo Hong) on reasonable request.

## Abbreviations

NSCLC: Non-small cell lung cancer
TMB: Tumor mutational burden
ROC: Receiver operating characteristic
AUC: Areas under the ROC curves
WES: Whole exome sequencing
CT: Computed tomography
TCGA: The Cancer Genome Atlas
TCIA: The Cancer Imaging Archive database
LUAD: Lung adenocarcinoma
LUSC: Lung squamous cell carcinoma
PACS: Picture Archiving and Communication System
DICOM: Digital Imaging and Communications in Medicine
ROI: Regions of interest
IHC: Immunohistochemistry
NGS: Next-generation sequencing
LASSO: Least absolute shrinkage and selection operator
VIF: Variance inflation factors
DCA: Decision curve analysis
GLCM: Gray-scale co-occupancy matrix
GLDM: Gray-scale dependence matrix
GLRLM: Gray scale run length matrix
GLSZM: Gray scale size zone matrix
NGTDM: Neighbouring gray tone difference matrix
PFS: Progression-free survival
OS: Overall survival
FDA: Food and Drug Administration
iCR: Immune complete response
iPR: Immune partial response
iSD: Immune stable disease
iCPD: Immune confirmed progression
